# Supplementary material for: The Molecular and Functional Characterization of Sensory Neuron Membrane Protein 1b (SNMP1b) from Cyrtotrachelus buqueti (Coleoptera: Curculionidae)
Source: Insects. 2024 Feb 4;15(2):111. doi: 10.3390/insects15020111 (PMC10889769; doi:10.3390/insects15020111)
Supplement: Supplementary file 1 [file insects-15-00111-s001.zip › insects-2826322-supplementary.pdf]

Table S1. Primer sequences used in this study.

Primer name	Sequences (5′-3′)
qPCR Primers for qPCR	
qSNMP1b-F	ACCGATATTCGCCACATTAC
qSNMP1b-R	ATTGTCTGGATCAAGACCCTC
qGAPDH-F	CGCTTCTGGTAACATTATCCCAT
qGAPDH-R	TCGACAACGGAAACATCGAC
Primers for prokaryotic expression [a]	
SNMP1b-F	GTGGACAGCAAATGGGTCGC**GGATCC**ATGAGAATAGGCATTCGTGACCAAG
SNMP1b-R	CAGTGGTGGTGGTGGTGGTG**CTCGAG**TTAGAAAATCAACTGGAGTTTCCTC

[a] The sites for restriction digest in primers for prokaryotic expression are indicated in bold.
